# Belonging for Women, LGBTQ+ and Black and Ethnically Minoritised People With Learning Disabilities: Findings From a Qualitative, Co‐Produced Study in England

**DOI:** 10.1111/1467-9566.70268

**Published:** 2026-10-15

**Authors:** Magdalena Mikulak, David Abbott, Kate Sykes, Poppy Budworth, Chris Hatton, Jess Mannion, Alex Kaley

**Affiliations:** ^1^ Division of Health Research Faculty of Health and Medicine Health Innovation One Lancaster University Sir John Fisher Drive Lancaster University Lancaster UK; ^2^ Norah Fry Centre for Disability Studies, School for Policy Studies University of Bristol Bristol UK; ^3^ School of Communities and Education Faculty of Health and Wellbeing Northumbria University Newcastle upon Tyne UK; ^4^ School of Environment, Education and Development (SEED) Department of Geography University of Manchester Manchester UK; ^5^ Department of Social Care and Social Work Faculty of Health and Education Manchester Metropolitan University Manchester UK; ^6^ Department of Social Science Faculty of Design, Education, and Social Science Ash Lane Atlantic Technological University Sligo Ireland; ^7^ School of Social Work and Sociology University of East Anglia Norwich Research Park Norwich Norfolk UK

**Keywords:** belonging, discrimination, intersectionality, learning disabilities, social determinants of health

## Abstract

Belonging is a key aspect of human experience. This qualitative study examined where diverse people with learning disabilities feel belonging, what shapes belonging and how it affects their health. Focus groups were held with people with learning disabilities across England who identified as women, LGBTQ+, and from Black and ethnically minoritised backgrounds. Twelve participants also took part in an additional data collection activity: a go‐along interview, ethnographic observation and/or photovoice. Our findings demonstrate shared themes and intersectional differences in participants' experiences of belonging. We emphasise belonging as embodied and as felt in diverse places and across time. It is also relational, open‐ended and facilitated by several actors and factors, which reflect the multiple social identities of participants. Experiences of nonbelonging are grounded in ableism, which, in turn, is compounded by and cannot be separated from racism, sexism and homo‐, bi‐ and transphobia. Through empirical examples, we highlight the relationship between belonging and health as bi‐directional and mutually reinforcing. To belong, people need to feel themselves authentically and valued in their complexity and uniqueness.

## 1 Introduction

People with learning disabilities^1^ experience less belonging (Bramston et al. 2002), poorer mental and physical health than their nondisabled peers and multiple barriers to accessing healthcare (Rickard and Donkin 2018; Sykes et al. 2024).

In England, men with learning disabilities die on average 23 years sooner than men in the general population. For women, the difference is 27 years (University of Bristol 2021). For Black people with learning disabilities, the median age of death is 40 years, 33 years for Asian groups and 30 years for mixed ethnic groups, compared with 62 years for White people with learning disabilities (Umpleby et al. 2023). Limited research with LGBTQ+ people with learning disabilities foregrounds their own experiences, with existing studies highlighting high levels of discrimination (Abbott and Howarth 2005). Women with learning disabilities face health inequalities which are often erased from discussion about gender and health inequities. They are less likely to be in work than men with learning disabilities (Nuffield Trust 2023) and their reproductive and parental rights are routinely overridden (Wiseman and Ferrie 2020).

Understanding how disability intersects with gender, ethnicity, sexuality and other axes of difference to shape experiences of embodied belonging is essential to understanding and addressing health inequalities among people with learning disabilities. Relatedly, multiple social identities, however tentative, can provide people with access to a range of group‐related resources and opportunities that can strengthen their sense of connectedness and support their health and wellbeing (Jetten et al. 2012; Haslam et al. 2018); yet, these factors are also rarely considered in relation to people with learning disabilities and belonging.

Feminist research has demonstrated that belonging is inherently intersectional and is constructed and negotiated in the interdependences of race, class, gender, ethnicity and other axes of social difference (Yuval‐Davis 2006, 2011)^2^. Within disability studies, intersectionality has proven a productive concept to challenge how ‘narratives of disability have both assumed and potentially subsumed the heterogeneity of lived experiences under the rubric of disability’ (Melbøe and Soldatić 2025, 447). Our data are England‐centric, but the lack of ‘intersectional imagination’ afforded to people with learning disabilities is a global issue. Wickenden writes (2023, 5) that global legal and rights‐based frameworks and organisations do not attend to the identity category of disability enough nor routinely challenge intersecting inequalities.

Belonging is a key aspect of human experience, but it is a perplexing term; it has been conceptualised as an emotional connection to place and people, characterised by feelings of security and being ‘at home’, while also encompassing the social, economic and political processes through which inclusion and exclusion are produced and contested (Yuval‐Davis 2011). Belonging is a complex process through which people negotiate multiple, and sometimes competing, identities across social locations. Social identities such as race, ethnicity, migration status or gender may serve to produce experiences of belonging and not belonging simultaneously and influence how belonging is claimed, recognised or contested (Gaither 2018; Martinussen and Lahiri‐Roy 2026).

‘Belonging’ has been critiqued as a concept that lacks clear definitions and theorisations (Fulton et al. 2021). In the absence of these—and perhaps also in opposition to them—a ‘weak theory’ approach to understanding belonging has been proposed (Wright 2015). Weak theory supports partial understandings and sees phenomena and ideas as open‐ended, entangled, interlinked and ‘in flux’ rather than demanding ‘comprehensiveness, exclusivity and grand claims’ (Wright 2015, 392). This approach allows researchers to work with the concept of belonging, generating understandings that enable us to attend

to the ways things, people, affects and places, with different trajectories, may come together, albeit in often tentative, inconclusive or evolving ways

(Wright 2015, 392)

Here, we work with understandings of belonging as affective, constituted through and by emotional attachments (Wright 2015) and embodied and in relation to material and spatial environments. Belonging and nonbelonging, and the space in‐between, are ‘felt’ on an emotional level, but they are also ‘sensed’ and imprint themselves in human bodies, supporting or undermining wellbeing (Mattes and Lang 2021). This understanding of belonging does not preclude us from appreciating that belonging carries different meanings for researchers and for people with learning disabilities. Strnadová et al. (2018) map the meaning of belonging for people with learning disabilities onto five interlinked themes: positioned belonging (related to cultural contexts people are born into and live), belonging as identity (related to how identities are formed and how ‘at home’ people feel with themselves), belonging as relational, belonging in relation to place (at local, regional and national levels) and belonging as a contributor to society. These themes are also reflected in our findings.

Belonging is shaped by wider structural and socio‐economic conditions that influence health (Michalski et al. 2020). Strong links exist between belonging and mental health (Gur and Bina 2023), including for people with learning disabilities (Kaley et al. 2022). Belonging also impacts physical health, influencing social connectedness, access to support and engagement with healthcare services (Clark et al. 2024).

Within learning disability studies, the affective and embodied dimensions of belonging have been foregrounded. Belonging is understood as a relational and subjective experience, involving feelings of acceptance, recognition, reciprocity and being valued by others (Gur and Bina 2023). This perspective moves beyond narrow notions of social inclusion and community participation to foreground people's embodied realities, offering a valuable lens for understanding how social environments shape health (Hall 2010). For people with learning disabilities, trusted relationships and community connections help them navigate complex and inaccessible health systems, facilitate communication with professionals and promote continuity of care. Structural barriers, including limited transport, fragmented services and reliance on time‐limited paid support, can restrict opportunities to build stable relationships, undermining belonging and health outcomes (Kaley et al. 2022).

Health inequalities research has often framed social isolation as an individual deficit, focusing on its negative health effects (Baah et al. 2019; Lynam and Cowley 2007), but inclusion alone does not guarantee belonging. Many community spaces remain exclusionary, requiring people with learning disabilities to adapt to normative expectations of communication and independence (Hall 2010). Such dynamics can reinforce feelings of people being ‘out of place’ and ‘out of sync’. Belonging allows us to think through social inclusion differently, shifting our focus ‘towards relationality and fluidity’ and centring an ‘intimate and informal inclusion’ (Reeves et al. 2023, 326).

This paper examines belonging, nonbelonging and spaces in‐between for diverse people with learning disabilities, and the impacts on their health and wellbeing. We attend to the relational, affective and embodied aspects of belonging, and to how these are spatially and temporally contingent and intensified through multiple axes of marginalisation, responding to stark intersectional differences in health outcomes for people with learning disabilities. This study examines how and where women, LGBTQ+ and Black and ethnically minoritised people with learning disabilities experience belonging, addressing important gaps in theory, policy and practice.

## 2 Methods

This study received ethics approval from the Faculty of Health and Medicine Research Ethics Committee at Lancaster University (Ref: FHM‐2024‐4720‐RECR‐2).

### 2.1 The Team and Approach

The team included seven academic researchers (from six universities in England and Ireland) and six community researchers^3^ (based across England, working at or supported by five self‐advocacy organisations). The team was comprised cis women and cis men. Community researchers identified as learning disabled and approached the research with their own diverse identities and experiences of learning disability and belonging; two identified as Black or from an ethnically minoritised background. The academic team identified as white, with some identifying as neurodivergent and/or disabled. The team worked together throughout the design, delivery and dissemination of the study and had been previously funded to identify research priorities, leading to the development of the ‘Belonging’ project. We adopted an inclusive, participatory approach to the research with everyone involved in producing and interrogating knowledge (Nind 2014; Charlton 1998). Bespoke training activities were developed and delivered to ensure that everybody had the knowledge and confidence to engage with the full range of research activities (Mikulak et al. 2022). Training included focus group co‐facilitation, research ethics, data analysis and writing for publication. Community researchers and their organisations were paid for their work. We met monthly online for general team meetings, in person on three occasions for more detailed work, more frequently online in small groups for data collection planning and data analysis and again in person at various stages to attend conferences, seminars and workshops.

### 2.2 Design

Participants could make decisions about how they wanted to take part. Most participants took part in a co‐facilitated focus group. Focus groups allow for participants to build on each other's ideas and experiences (Mietola et al. 2017). Participants could opt for a 1‐1 interview instead of a focus group for reasons such as accessibility, travel and comfort. In addition, some participants were invited to take part in ‘go‐along’ interviews, ethnographic observations and/or photovoice. Including elements of ethnographic observation allowed for ‘being with’ participants in places that mattered to them (on the value of ethnographic methodologies for research with people with learning disabilities, see Grace et al. [2024]); this going to and being in places also gave us an insight into the embodied dimension of belonging. Using a variety of qualitative methods offered flexibility and adaptability, which accounted for participants' and researchers' access needs and preferences (Budworth 2023).

### 2.3 Sampling and Recruitment

Focus groups or 1‐1 interviews were conducted with LGBTQ+, Black and ethnically minoritised and women with learning disabilities. Participants were identified through self‐advocacy and community organisations and personal and professional networks of the research team. These relationships offered participants extra support before, during and after participating; community organisation staff could offer advice on what to expect from participating and help with scheduling. Organisations supported participants in choosing a focus group (e.g. approaching members who identify as LGBTQ+). Participants who were eligible for more than one group took part in only one, although they often discussed their overlapping identities within that group.

We developed accessible recruitment materials in easy‐read format, including participant information sheets, consent forms and topic guides for focus groups and ethnographic observations. All participants provided consent (either written or verbal) before taking part.

About 41 people took part, from nine locations across England: 13 people in the women's groups; 14 people in the Black and ethnically minoritised groups; and 14 people in the LGBTQ+ groups. Across the three groups, participants identified their pronouns as: he/him (*n* = 16), she/her (*n* = 22), they/them (*n* = 2) and no pronouns (*n* = 1). Age was reported by 34 participants; it ranged from 19 to 71 (mean: 39 years).

### 2.4 Data Collection

Data collection took place from March to September 2025. Each focus group was conducted by one academic researcher and a community researcher (in six of the nine cases). A supporter (e.g., paid staff, family and friends) could join the participants. Participants were given a £25 gift voucher. Travel costs were reimbursed.

Participants were first given various creative materials and invited to create artwork on the theme of belonging. Photographs were taken for analysis, enabling participants to keep their creations. Participants were then asked about belonging in audio‐recorded focus groups or a 1:1 interview. Up to two participants per focus group were invited to take part in an additional research phase. Either choosing from a go‐along interview, photovoice of places where they felt they belonged and/or an ethnographic observation at an event or place. Twelve participants were involved in follow‐up activities, choosing a variety of locations including workplaces, music venues, shopping centres and walking routes. Audio files were transcribed verbatim by a professional transcription service. All data were anonymised before analysis.

### 2.5 Data Analysis

The flexible approach to data collection resulted in a rich data corpus allowing for triangulation and analytical depth. Each dataset was analysed separately, then insights and interpretations were brought together iteratively.

We adopted a participatory approach to analysis (Nind 2011) and drew on/adapted the Creative Collaborative Data Analysis method, developed through a previous co‐produced project with researchers with learning disabilities (Mannion 2024). We drew on content analysis (Grbich 2013), collaborative interpretation of findings (Mannion 2024) and embodied data analysis (Thanem and Knights 2019).

We used content analysis to reduce and organise the data. During content analysis, textual data were systematically categorised by identifying patterns in the words used (Grbich 2013). Word frequency was explored in NVivo by identifying the most used words in the transcripts, excluding functional words (e.g., ‘the’ and ‘a’). The most frequently used word was ‘belonging’, followed by ‘feeling’. A researcher then selected all data relating to ‘belonging’ and presented it in an accessible document for the research team, repeating this process for the word ‘feeling’. Three online data analysis sessions were held, each lasting 1.5 hours. Each session included the community researchers who had led the focus groups, their supporters and academics. The group reviewed and refined the developing analysis, drawing on the community researchers' first‐hand knowledge of the focus group discussions they had facilitated alongside the collective expertise of the wider research team. All discussions were recorded and transcribed. The researcher then produced an accessible summary outlining the team's developing analysis. Groups met once more to discuss the themes and interpretations formed in the process. To strengthen robustness and ensure that the depth of data was captured, focus group and interview transcripts were also analysed and coded thematically in NVivo by the first author (Braun and Clarke 2019).

The next stage focused on the creative data and drew on the embodied data analysis (Mannion 2024). We held an in‐person analysis workshop, where the artwork (or its photographs) were displayed around the room and organised into the three groups of participants. Together, we moved around the room viewing and discussing the artwork. We recorded our thoughts and reflections on sticky notes and placed them alongside the relevant artworks. We then discussed what stood out to us and considered similarities and differences, and what these might mean. These discussions were documented by a note taker.

Finally, ethnographic observation notes and go‐along interview transcripts were coded and thematically analysed in NVivo by two academic researchers (Braun and Clarke 2019). Themes from this dataset were compared with the themes from focus group and interview data. The themes and insights from the divergent datasets were brought together, contrasted and compared by the team.

## 3 Findings

Findings reflect themes identified within the data, beginning with intersectional differences in participants' understanding and relational experiences of embodied belonging. We then discuss the diverse range of places, actors and factors that impact belonging, highlight its affective dimension and report on participants' understanding of the relationship between belonging and health, including strategies for resistance in the face of compounded discrimination.

Information is provided to show quote sources—from a focus group or interview (FG; IN), ethnographic observations or follow‐up interview (FUI) and the type of focus group: women [W], ethnic minority [EM] and LGBTQ+. Thus, ‘(FG_EM_1)’ denotes the first focus group involving ethnically minoritised people with learning disability. Finally, ‘alt’ denotes an alternative format, for example, a participant choosing to write their answers instead of speaking. We use pseudonyms for all participants.

### 3.1 Experiences and Feelings of Belonging

For all participant groups, belonging was linked to positive affect, or feelings, such as ‘You feel welcomed’ (Arlo_FG_EM_1), ‘Feel, feel safe’ (Yusef _FG_EM_1) and ‘Feel happy’ (Maya_FG_EM_1).

Family, friends and partners, and doing things together, gave people a sense of belonging, highlighting its relational aspect:

Facilitator Who helps you feel like you belong?

Martha My family, my boyfriend, and my friends, and [day activities group] (FG_W_1alt)

It’s easier for me to express my emotions when I am with my friends

(Guppy, FG_W_1)

Reciprocal relationships of care helped people feel like they belonged, regardless of biological familial ties:

My auntie’s sister‐in‐law (…) she’s kind and she really understands, and she loves me as a daughter (…) and sometimes I’m there for her as well (…) I love her like an auntie as well

(Maria, FG_EM_2)

Self‐advocacy groups (run by people with learning disabilities with support) were key in supporting belonging and fostering connections:

Yusef and Martin tell me that they have been friends for 15 years and first met at a disabled people’s group who then put them in touch with a self‐advocacy group for people with learning disabilities, both organisations of which they are still part of now

(Ethnographic notes, FUI_EM_1)

Self‐advocacy connections gave people access to other places where they could experience belonging:

Facilitator So how come you haven't been before [to Pride], and why did you decide to go this year?

Rowan Because I needed someone to go with…. Going on your own is never something I would do.

Facilitator Yeah, so who did you go with this year?

Rowan Uh, a couple of people from the LGBT group.

Facilitator Is that the [Self‐Advocacy group] LGBTQ [group]?

Rowan Yeah, yeah, yeah (IN_LGBTQ+_3)

Although family was an important site of belonging for participants, there were sometimes tensions and contradictions around family and belonging that benefitted from an intersectional analysis. Some participants highlighted discrimination and nonbelonging within families:

When I was little my cousins (…) used to take my hearing aids out and joke (…) now I don’t keep in touch with them. Still, cause they still joke, they’re old and (…) they have their own children. Their children start doing this, so I don’t, I don’t keep in touch with them… I was only small, [they would] take my hearing aids and throw it in the toilet

(FG_EM_1)

For LGBTQ+ participants, there was the additional risk of being rejected by their family based on their sexuality or gender identity:

I’ve got one sister who's against me, against me, against because I am gay, because her Christian [faith] and that

(Ell, FG_LGBTQ+_1)

Being able to come out and be accepted by everyone in the family could be a key moment of affirming belonging:

My family knew I was lesbian before me (…) So like for me to try and tell them, they were like ‘oh, we already know ‘(…) the only one person that didn’t know was obviously my dad, and I was thinking OK, how, how is this, you know, gonna pan out? (…) So I was really emotional about it, and dad said, ‘You know like it’s fine, like I’m not gonna love you any less.’ And that for me felt really belonging for me

(Lucie, FG_LGBTQ+_2b)

As well as human connections, some participants shared experiencing belonging with nonhuman companions ‘My dog! (…) She likes cuddles, and she comes up to you and likes to, she knows when you are not very well’ (Florence, FG_W_1). For some, inanimate objects, like teddies, supported belonging and provided a safe way to explore their multiple identities:

I have teddies at home (…) I made them a gay couple (…) When I was experimenting with, oh I wonder what it’s like to be this and that’s why I have done that I realise. But they love each other, its great

(Jamie, FG_LGBTQ+_1)

Most participants felt a sense of belonging in their homes. However, this was problematised in the focus groups with women, where questions of choice over where to live and desires for more independence were raised. This could relate to wanting to move out from the family home or move out from an unsuitable shared home.

I live with my mum and dad, but I want to live on my own independently

(Susie, FG_W_1)

Ivy I feel that I want to move, and if I… I don't want to try and upset me sisters and brothers …I don't know how to go about it.

Facilitator Are you happy where you're living now?

Ivy Well… No, not really, but I have to be (FG_W_1).

### 3.2 Intersecting Experiences of Nonbelonging

Intersectional, embodied experiences of marginalisation undermined belonging for all groups of participants. For Black and ethnically minoritised people, the compounded effect of racism and ableism was significant:

I got into a very top drama school (…) The culture just didn’t seem right… The way my disability was perceived (…) How they perceive ethnic minorities in particular (…) I actually went through so much challenges there, which kind of pretty much left me as a broken individual

(Ravi, FG_EM_2)

In the LGBTQ+ groups, exclusion based on learning disability was compounded by homo‐, bi‐ or transphobia:

it is tough for both groups, but I think at the end of the day LGBT [community] gets a lot of shit and it’s even worse if you have a disability

(Jayden, FG_LGBTQ+_1)

LGBTQ+ people with learning disabilities can also experience discrimination based on others making assumptions about their (in)ability to know their gender identity and sexuality:

I think a lot of people think we don’t understand, and in reality we do. We know our feelings, we know our identities, it’s just they don’t like to think we are capable of it

(Jayden, FG_LGBTQ+_1)

For trans participants, changes in legislation around trans people's access to toilets were an additional source of anxiety:

I was worried at work (…) That’s the only one that really panicked me (…) When it [the law] also came into place… I use the men’s, unless my disability means I need the disabled

(Phoenix, FG_LGBTQ+_2b)

LGBTQ+ people with learning disabilities can experience ableism within the LGBTQ+ community. Not disclosing one's disability was a way to cope with this, which is not an option for visibly, physically disabled participants:

Ell I don't tell them when I go in bars that I've got a learning disability. They'll walk away when you say that then […]

Jayden It's a bit harder…

Ell They walk away from you.

Jayden It's a bit harder for me, err, because they can see I have a [physical] disability (FG_LGBTQ+_1)

Experiences of ableism from within ‘one's community’ were also reported by participants in the ethnically minoritised group, particularly in relation to keeping up with the ‘faster’ pace of nondisabled peers:

When I used to go primary school, the Asian community didn’t, didn’t, didn’t understand my disability… Cause I was a bit slower. The teacher had to repeat the second time then I understood…. when the children need to go home, the parents used to chat to my mum, ‘Why’s your son not as fast as my son? He always keeps the class behind’

(Yusuf, FG_EM_1)

Participants in the women's group shared explicit experiences of gender‐based harassment:

I’ve been followed by a guy once. He kicked my dog and he kept on stalking me trying to catch me alone, and waiting around the corner… so my mate will walk me down the street, and they saw the guy waiting for me, but he didn’t come near me cause my mate there

(Keira, GF_W_3)

These experiences, alongside other instances of compounded discrimination, compromised women's sense of safety, undermining belonging.

### 3.3 Places of Belonging

Participants reported a diverse range of places they felt belonging. Examples included gay bars, queer events, on the beach, in the local park, in pubs, with day activities groups, at home, places of worship, at punk and metal gigs, in the city, at work and more. These places reflected participants' complex identities and ways belonging is embodied:

I… I belong, as I said, on the road […] I don’t, as any Traveller will say, I don’t like four walls and door. I prefer a set of wheels underneath me

(Max, FG_LGBTQ+_2a)

Affective aspects of belonging were visible in feeling in or ‘out of place’; living in a particular place could support or undermine a person's sense of belonging. For example, for Black and ethnically minoritised people, living in an area that was not diverse could make belonging harder:

Maya I moved here… It's alright, not multicultural like [multi‐ethnic inner city ward] (…) it's a bit awkward, cause everybody keeps themselves to themselves, so obviously nobody will talk unless they have to, innit? (…) it's not that diverse.

Facilitator (…) Would you like it to be more diverse?

Maya Yeah (FUI_EM_2).

In the focus group, Maya shared that she spends 'time mostly at home’ with her family and that she does not feel she fits in her local community: ‘when I go out to places people don't understand where I'm coming from so it's a bit awkward, so I stopped going to the places’ (FG_EM_1). Maya experienced feelings of not fitting in and awkwardness also in college and at community centres, which left her questioning herself: ‘I stopped going, cause sometimes when they both don't understand me, I was thinking oh, is it me?’ (FG_EM_1).

Moving to a place that felt more welcoming and accepting meant people were able to feel safer, more at ease, which helped their sense of belonging. Ell moved from a smaller city into a bigger one, known for its LGBTQ+ culture:

Facilitator 1 So what about [current city] made you feel more safe, Ell?

Ell Be what you are.

Facilitator 2 So you can be yourself sort of thing.

Ell Yeah.

Facilitator 1 And did you feel you couldn't be yourself in [previous city]?

Ell No. (FG_LGBTQ+_1).

Local initiatives fostered connection and provided an infrastructure for belonging. Gig buddies featured prominently in the LGBTQ+ focus groups, giving people access to events and spaces they enjoyed more frequently:

Facilitator So before you had a Gig Buddy did you go to concerts?

Ell I went to concerts in [town] by myself, and I saw T‐Rex and other artists…

Facilitator Did you go as often? Do you go as much as you do now?

Ell I go more now. (FG_LGBTQ+1).

When local initiatives disappear, it impacts belonging negatively and, indirectly, health. Maya used to attend a local walking group which has stopped due to decreased interest.

Facilitator So what's your favourite memory from the walking group?

Maya Walking down this road, and then walking to see the trees. That's my favourite memory (…) And now I'm just lazy, [inaudible] where I live there's nothing, shall I say, nothing to do for like adults or teenagers, nothing… [I got told to] exercise at hospital this week. That's what the nurse told me to do, lose weight (FUI_EM_2)

### 3.4 Community Connections

Religion was described as a source of belonging, connecting people to communities and places:

River Um, there's a few friends that've accepted me for who I am.

Facilitator And how did you meet these friends?

River Uh, at church.

Facilitator At church?

River Yeah, I met ’em and then come out to ’em, and then they're like ‘tell us how we can help’, which is quite nice (FG_LGBTQ+_2).

Facilitator Why do you feel that you belong in the mosque? [interpreter translates for Dawar]

Interpreter He [Dawar]'s a Muslim and he likes to pray, so he goes there to pray and he feels comfort and belonging there in his religion (FG_EM_3)

Culture can act as an anchor for a person's belonging. Ravi shared that listening to Afro Beats helped him feel belonging (FG_EM_2); for LGBTQ+ participants, drag shows and TV shows (e.g., Heartstopper) were important points of shared reference.

An embodied sense of belonging can also be accessed through shared interests with more fleeting communities. Experience of being physically supported by a fellow fan at an event illustrates how moments of embodied belonging and joy are reflective of the ‘intimate and informal inclusion’ (Reeves et al. 2023):

I went to Slam Dunk [punk, metal, and alternative music festival] one year, I was near the front cause it was an artist I really like, and then the mosh pit started, I was like shall I back away? Cause I’ve got cerebral palsy and I fall over, and so I thought I’ll stand here and see how I manage, and then said to the person next to me I’m gonna have to be careful cause I’ve got cerebral palsy, and they were like I’ll hold you if you want, and then I was able to join in in the mosh pit cause I was being held by… Held up by people! (…) Someone I’d never met before was like well let’s make this accessible

(Phoenix, FG_LGBTQ+2b)

Similarly, Lucie shared how she feels she both belongs but also feels inspired through horse riding:

I also love going to like horse shows as well, because I feel like there is a lot more of the equine community, which is really lovely (…) I’ve met a few of my favourite Paralympic riders as well (…) Which they’ve obviously inspired me a lot, so, and I feel like I belong with them as well, which is really nice, because they, they feel like a little family (…) like obviously it kind of grows [one’s] support network

(Lucie, LGBTQ+_2b)

Having the right support is key to fostering belonging, as highlighted by the exchange with Bruno:

Bruno Yeah, I've only got CDs [of Slayer], I've never seen them (…) so when you go to a gig, do you go alone, do you go with someone else?

Phoenix Um, if it's in [town] I'll go alone. If it's anywhere else I'll go with support (…) I have a PA who, he is into the same music anyway, so… He'll be wanting to go, so he might as well go with me (FG_LGBTQ+2b).

Access to events, such as Pride, that might support a person's sense of belonging can be contingent on material resources.

I haven’t decided (…) It depends if I can get cheap tickets (…) It’s about £60 something like that (…) And also what takes the piss, excuse my language, is that the drinks and food is expensive as well

(Jayden_FG_LGBTQ+_1)

Online communities can offer people additional spaces of belonging, particularly evident in online gaming. When asked if gaming was his ‘hobby’, Bruno insisted it is much more than that. He shared: ‘To me it's life really… Gaming is like my family’ (Bruno, FG_LGBTQ+2b). For LGBTQ+ people, the gaming community was important also in terms of coming out and feeling accepted:

It’s a big gaming community (…), all the people that I know accepted me as, as I come out

(Max, FG_LGBTQ+_2a)

Technology can support a person's belonging offline, helping to stay in touch with family and friends. Liam shared that he enjoys it when his family sends him texts: ‘I like to hear what's going on (…) back at home. (…), I know they're alright’ (FG_EM_2).

For another participant, who shared he feels like he belongs in the city he lives in, technology helped him physically explore new places:

I like to go new places. Like last week I went to [suburb], walked around the park and then got the bus back home (…) I have this app called City Map[per] (…)it’s an app where you can, to help you travel to places (…) it’ll tell you like the route, the routes, the buses and how long, like how long the buses will take and stuff

(Arlo, FG_EM_1)

Technology also helped people handle money, with Tambah sharing about Apple Pay: ‘you can pay for everything, you can pay on the train, you can pay for travel’ (FG_EM_1). However, for people who relied on cash, technology creates barriers:

When looking to get some lunch together (…) we walk to the food hall (…) some places only take card now, which proved difficult as Yusef had only brought a £10 note with him. I asked if he ever uses card, and he said he used to but would often forget the pin and so opts for cash now

(FU1_EM_1)

### 3.5 The Belonging–Health Relationship

There was a clear and consistent connection between belonging and wellbeing. This was at times contrasted with the challenge of finding places and people where belonging was felt/sensed:

There’s been very, very little, and very few organisations where I have been belonging. (…) So, so, so when you belong somewhere it does feel great, but when you obviously don’t belong somewhere, and you’re trying to search for it, that can be quite exhausting

(Ravi, FG_EM_2)

It [belonging]’s good because it’s good for your mental health, but sometimes you have to figure out where you belong in this world… you might not know where it is at first (…) I am resilient, I don’t give up

(Guppy, FG_W_1)

Poor mental health undermines belonging:

Facilitator Is there anything that stops you feeling like you belong? (…)

Guppy A nice and easy question… My mental illness (…) it takes partially some of my independence away from me. Because it is not easy managing an illness that isn't nice to you (FG_W_1).

Physical health issues can be a barrier to doing things that help belonging. In response to Guppy above, Florence commented:

I got the same thing, I am annoyed because I can’t really do much things because my stabbing pains (…) I like to do more things, really

(Florence, FG_W_1)

### 3.6 Resisting Compounded Marginalisation

When reflecting on health and belonging, some participants emphasised that belonging for them was not about conforming or ‘fitting in’ but rather about being authentically themselves:

I might be quite neutral on it [belonging being good for health], cause I don’t care too much if I don’t fit in, cause I just won’t go to the place, I’m happy with like myself (…) I won’t look for the place that I fit in for, I’ll just be myself and see what comes to me and sticks to me (…) I don’t alter myself

(Phoenix, FG_LGBTQ+_2a)

Trying to fit in can be exhausting and have a profoundly negative impact:

On some times it can be that people just, just don’t want to be inclusive with you, or there’s a sense you have to be a certain type of person in order to fit in, which I refuse to do (…) I mean I tried it before, it backfired on me and I ended up suffering a bit of a breakdown from that

(Ravi, FG_EM_2)

It is in this context that staking one's claim to belonging becomes an act of defiance and active resistance in the face of exclusion:

I know people can be cruel with people what’s got disability and everything, so that can stop you [feeling like you belong], if someone says summut nasty to you (…) Cause I got a couple friends, like people have said summut nasty to them and they’ve felt like doing summut silly, like ending their lives, and I went, ‘Don’t be silly, that means they’ve won the battle. You do not let them win; you win the battle… You show them you do belong’

(Keira, FG_W_3)

Intersectional experiences of belonging can act as a source of positive affect, pride and resilience.

Facilitator So then if you think about when you do belong, what kind of feelings come up then?

Jamie You feel happy, safe and accepted. You're not worried. I belong here. I belong in the LGBT community.

Jayden I am pretty proud to be part of it quite honestly (FG_LGBTQ+_1).

For some participants, this meant feeling part of a wider struggle for rights and social justice. Max, a member of the Traveller community, explained: ‘There is the Travellers Right movement […] We are, as we said, fighting back [against discrimination]’ (Max, LGBTQ_2a).

## 4 Discussion

We aimed to explore how and where a diverse range of people with learning disabilities experience belonging. We sought to problematise a tendency within research, practice and policy to treat people with learning disabilities as a homogeneous group (Kover et al. 2024). Our findings demonstrate that belonging is relational, embodied and fundamentally shaped by intersecting identities across gender, race and sexual orientation.

A central finding of this research (consistent with other research) is that belonging supports mental health and physical wellbeing, whereas poor physical and/or mental health undermines people's belonging. These findings position the relationship between belonging and health as bi‐directional and mutually reinforcing. Regardless of gender, ethnicity or sexual orientation, belonging was associated with feelings of happiness and wellbeing, reduced anxiety and feelings of self‐worth. Belonging is an important determinant of health (Kafer 2013) and should be central to thinking about and addressing the health inequities experienced by people with learning disabilities.

Belonging was undermined by intersecting experiences of discrimination (ableism, racism, sexism and homo‐, bi‐ and transphobia). The effects of this compounded discrimination were shared across groups and were experienced as feelings of withdrawal, exhaustion and overall harm to mental health (Haim‐Litevsky et al. 2023).

Similarly to Strnadová et al. (2018), we have found that belonging is about having relationships. Interpersonal relationships provide the core infrastructure for belonging—all groups of participants described how belonging was facilitated through friendships, romantic relationships, ‘chosen’ family, peers (i.e., self‐advocacy groups) and nonhuman companions. Although the nature of these relationships varied, the importance of caring, reciprocal and meaningful relationships was universal. Few participants saw formal health or social care services as sources of belonging. Rather, services became supportive in this regard only when they enabled access to other spaces (i.e., cultural events, LGBTQ+ venues, nature), facilitating belonging in relation to place and/or positioned belonging (Strnadová et al. 2018). Overall, this study highlights that belonging for people with learning disabilities is complex and evolving, supported by affective attachments to places and negotiated with other people. Belonging is not a fixed emotional response to feeling ‘in place’, or ‘at home’, but a dynamic, multi‐layered, relational, embodied and intersectional process (Yuval‐Davis 2006, 2011). Applying an intersectional lens acknowledging multiple axes of difference enables us to comprehensively consider the vast heterogeneity within the learning‐disabled community (Brinkman et al. 2023). It allows us to unpick how sites commonly associated with belonging, such as families, can also be marked by exclusion: by experiences of ableism, or as is the case for LGBTQ+ people with learning disabilities, experiences of homo, bi‐ or transphobia. For women with learning disabilities, belonging may be further contrained by gendered expectations and lack of autonomy.

In line with previous work, this research has identified that for people with learning disabilities to feel they belong, they need to feel safe and happy (Kaley et al. 2022), but we also found that they need to feel themselves authentically and valued in their complexity and uniqueness. These reflections build on the work done by others on belonging as identity (Strnadová et al. 2018), aligning also with more imaginative—‘crip’—understandings of belonging, which stretch ideas about community and relationships rather than making people ‘fit’ with ableist norms (Kafer 2013). Crip theory builds on queer theory and disability activism, challenging normative ideas about dis/ability, bodies and sexuality that, in turn, shape whether people belong or not. Shifting away from ideas of normal/abnormal, crip theory centres the lives and experiences of disabled people and communities, positioning them as valuable in society and not a medical problem to be fixed or eradicated (Mcruer 2006; Kafer 2013).

Participants were not passive recipients of ‘labels’ but actively negotiated their multiple social identities, making choices about which identity to foreground and with whom. In broader disability research, there is a recognition of intersectionality as an ‘agential strategic practice’, whereby an identity category is asserted, or downplayed, in relation to constraints of the socio‐structural relationships of power. This ‘enables structurally oppressed community members to prioritise one identity above another’ to ‘gain recognition, access to greater social resources and capital, and open opportunities for inclusive participation within social, political, cultural, or economic institutions’ (Melbøe and Soldatić 2025). We demonstrate how people with learning disabilities actively negotiate their multiple identities and make strategic choices and claims to belonging, through anchoring themselves in social identities beyond that of learning disability. This is made visible through active resistance to normative assumptions about disabled (‘crip’) and queer lives (Kafer 2013), for example, through challenging assumptions that disabled people do not understand or are unable to affirm their gender identity and/or sexual orientation. Similarly, insisting on one's right to belong in a society, in which one faces discrimination and marginalisation, becomes an act of defiance in the face of social injustice.

Belonging happens at many different sites, including in digital spaces. This points to both opportunities for thinking about belonging of people with learning disabilities in more open‐ended ways, and to the need to explore how accessible and diverse these sites are. Technology and online communities offer exciting possibilities to connect with others, as well as support staying in touch with family and friends, and people's autonomy. However, questions of digital exclusion and the need for support to navigate the online world continue to impact people with learning disabilities (Mikulak et al. 2023; Chadwick et al. 2022).

A sense of belonging to one's neighbourhood and local environments matters for people's ability to access and engage in social activities (Clark et al. 2024). Our research highlights how racial, geographic and socio‐economic disparities in belonging for people with learning disabilities also determine who benefits from the social and economic aspects of communities and services. Our research also reveals additional needs related to people's intersecting identities; for example, ‘safe spaces’, which, for LGBTQ+ participants, meant the availability of queer‐affirming spaces that are also welcoming to people with learning disabilities, who might also be physically disabled.

In line with previous research (Strnadová et al. 2018), ableism was a barrier to belonging for all groups of participants. It has been highlighted that, for positive change to happen, there is a need for nondisabled people to have increased, direct engagement with disabled people. However, people with learning disabilities ‘may struggle to influence society's perception, and that challenging negative attitudes is a shared social responsibility that should not rest entirely on stigmatised groups’ (Simmons et al. 2026). As our research shows, looking for places and people to belong with can be exhausting, particularly for Black and ethnically minoritised people, and refusing to ‘fit in’ at all costs can be a valid response that is necessary to protect one's mental health. Our research also highlights the resilience and creativity with which people with learning disabilities challenge discrimination when they stake their claims to belonging, against the odds.

There is strong evidence that self‐advocacy groups have positive impacts on the wellbeing of people with learning disabilities (Tilley et al. 2020). Our research confirms this while also highlighting the value of self‐advocacy groups that bring together people based on particular social identities, providing them with a safe space to belong and to discuss their specific challenges and experience shared joy (e.g. a ladies' night out, or a drag show).

Finally, there is a need for services to take on the work of supporting the belonging of people with learning disabilities. This requires a shift from viewing people through a single social identity to understanding how people inhabit and move between multiple identities and how services might facilitate access to people, places and events where belonging can be experienced. Importantly, people with learning disabilities may need additional support to manage the complexities of navigating different aspects of their identities. For example, a person may need support to balance normative expectations from family and their LGBTQ+ identity. Similarly, negotiating autonomy alongside gender and ableist norms that equate femininity with dependency requires input from supporters—not in ways that undermine women's agency, but rather to support them in articulating and asserting their preferences about where and how they want to belong. Experiences of racism demand that services are set up and run to challenge, not compound these; allyship from supporters is key. This also requires reframing support as belonging work, not merely practical assistance with everyday tasks or activities. It is key that any such work remains sensitive to intersectional differences, avoids making assumptions about people's identities and leaves room for exploration and people's agency.

## 5 Conclusion

This study offers an original, theoretical contribution to disability scholarship conceptualising belonging for people with learning disabilities as an embodied, intersectional, relational and spatially and temporally negotiated process shaped by ableism, racism, sexism, homo‐, bi‐ and transphobia. This framing offers a lens that can inform research, practice and policy in the United Kingdom and beyond, as it responds to calls for placing intersectional equity at the centre in health equity work (World Health Organisation 2022).

Although homogenised accounts of belonging for people with learning disabilities offer some general insights into what helps or prevents people from belonging in some contexts (Gur and Bina 2023), by ignoring intersectional differences, they inevitably fall short of providing meaningful insights into unique challenges and opportunities people with learning disabilities face when negotiating embodied belonging as women, members of an ethnically minoritised group or LGBTQ+ people. Importantly, intersectional sensitivity should not stop us from remaining vigilant of the risk of essentialising people's identities and experiences. It is in taking the weak theory approach that we can remind ourselves of the always‐tentative nature of our claims to knowledge, and of the need to remain attentive to how belonging is an open‐ended, always in the making, and remaking, concept. Our study, for example, did not recruit people with higher levels of support needs and/or people who communicate without words. This is a point of intersection left out of most research—including ours.

Finally, how we study belonging matters. Methodologically, our research aligns with Kover et al.’s (2024) point that there is a symbiotic relationship between research approaches and attempts to tackle enduring inequalities. Our co‐produced research with people with learning disabilities demonstrates how belonging operates as an important, yet understudied, social determinant of health, as an embodied practice and as a key site of agency and resistance; it also highlights that belonging remains a key concern and research priority for this marginalised group.

## Author Contributions

**Magdalena Mikulak:** funding acquisition, conceptualization, project administration, formal analysis, writing – original draft, writing – review and editing. **David Abbott:** conceptualization, funding acquisition, formal analysis, writing – original draft, writing – review and editing. **Kate Sykes:** conceptualization, formal analysis, writing – original draft, writing – review and editing, investigation. **Poppy Budworth:** investigation, formal analysis, writing – original draft, writing – review and editing. **Chris Hatton:** conceptualization, formal analysis, writing – review and editing, writing – original draft. **Jess Mannion:** investigation, formal analysis, writing – review and editing. **Alex Kaley:** conceptualization, formal analysis, writing – review and editing.

## Funding

This research is funded by the National Institute for Health and Care Research (NIHR) School for Public Health Research (SPHR) (Grant Reference Number NIHR 204000) and NIHR School for Social Care Research (SSCR). The views expressed are those of the author(s) and not necessarily those of the NIHR or the Department of Health and Social Care.

## Ethics Statement

This study received ethics approval from the Faculty of Health and Medicine Research Ethics Committee at Lancaster University (Reference: FHM‐2024‐4720‐RECR‐2).

## Conflicts of Interest

The authors declare no conflicts of interest.

## Data Availability

The data are not publicly available because of privacy and ethical restrictions.

## Endnotes

^1^
‘Learning disability’ is the terminology conventionally used in UK policy, practice and research contexts. In other contexts, the equivalent term is ‘intellectual disability’.

^2^
The concept of intersectionality originated in the Black feminist scholarship of Kimberle Crenshaw (Crenshaw 1989), developed to account for the distinct and compounded ways racism and sexism impact the lives of Black women. Intersectional research on belonging continues to generate important insights (Subramani 2024).

^3^
‘a member, and has direct experience of, a particular community and uses this knowledge to facilitate the gathering and interpretation of data’ (Salway et al. 2015). Researching health inequalities with community researchers: practical, methodological and ethical challenges of an ‘inclusive’ research approach. *Research Involvement and Engagement*, 1, 9.
